# A ketogenic diet as a potential novel therapeutic intervention in amyotrophic lateral sclerosis

**DOI:** 10.1186/1471-2202-7-29

**Published:** 2006-04-03

**Authors:** Zhong Zhao, Dale J Lange, Andrei Voustianiouk, Donal MacGrogan, Lap Ho, Jason Suh, Nelson Humala, Meenakshisundaram Thiyagarajan, Jun Wang, Giulio M Pasinetti

**Affiliations:** 1 Neuroinflammation Research Laboratories, Department of Psychiatry, USA; 2 Department of Neurology, Mount Sinai School of Medicine, One Gustave L. Levy Place, Box 1668, New York, New York 10029, USA; 3 Bronx Veterans Affairs Medical Center, 130 W Kingsbridge Road, Bronx, NY 10468, USA

## Abstract

**Background:**

The cause of neuronal death in amyotrophic lateral sclerosis (ALS) is uncertain but mitochondrial dysfunction may play an important role. Ketones promote mitochondrial energy production and membrane stabilization.

**Results:**

SOD1-G93A transgenic ALS mice were fed a ketogenic diet (KD) based on known formulations for humans. Motor performance, longevity, and motor neuron counts were measured in treated and disease controls. Because mitochondrial dysfunction plays a central role in neuronal cell death in ALS, we also studied the effect that the principal ketone body, D-β-3 hydroxybutyrate (DBH), has on mitochondrial ATP generation and neuroprotection. Blood ketones were > 3.5 times higher in KD fed animals compared to controls. KD fed mice lost 50% of baseline motor performance 25 days later than disease controls. KD animals weighed 4.6 g more than disease control animals at study endpoint; the interaction between diet and change in weight was significant (p = 0.047). In spinal cord sections obtained at the study endpoint, there were more motor neurons in KD fed animals (p = 0.030). DBH prevented rotenone mediated inhibition of mitochondrial complex I but not malonate inhibition of complex II. Rotenone neurotoxicity in SMI-32 immunopositive motor neurons was also inhibited by DBH.

**Conclusion:**

This is the first study showing that diet, specifically a KD, alters the progression of the clinical and biological manifestations of the G93A SOD1 transgenic mouse model of ALS. These effects may be due to the ability of ketone bodies to promote ATP synthesis and bypass inhibition of complex I in the mitochondrial respiratory chain.

## Background

Amyotrophic lateral sclerosis (ALS) is an adult-onset neurodegenerative disorder in which spinal and cortical motor neurons die causing relentlessly progressive weakness and wasting of skeletal muscles throughout the body [1]. Affected individuals usually die within 3–5 years of symptom onset [2]. The cause of neuronal cell death is uncertain. Loss of oxidative control, generation of excessive oxidative free radicals, neurofilament accumulation, excitotoxicity and mitochondrial membrane dysfunction are implicated in the pathogenesis of ALS [3,4]. Apoptosis is thought to be mechanism of cell death [2].

ALS is an inherited disorder in 10% of patients (familial ALS, FALS) [1]. Mutations in the gene encoding the enzyme Cu/Zn superoxide dismutase 1 (SOD1) was the first mutation identified to be associated with FALS [5]. Transgenic mice with the mutant human SOD1 transgene have a disease similar to human FALS characterized by progressive weakness of muscles eventually leading to death from respiratory muscle weakness and respiratory failure [6]. How mutations in the SOD1 gene result in ALS is unknown. However, the FALS syndrome results not from a loss of SOD1 function but rather a toxic gain of function [2].

Mitochondria may play a critical role in the pathogenesis of ALS and is perhaps a target for the mutant SOD1 protein [7]. Mutant SOD1 has been localized in the mitochondria binding bcl2, the cells primary anti-apoptotic protein [8,9]. Decreased mitochondrial complex I activity has been measured in platelets [10], biopsied muscle and the spinal cord of ALS patients [4,11,12]. In cultured neurons treated with pharmacologic agents that block complex I, addition of ketone bodies restores complex I function [13].

Because of the ability of ketones to alter mitochondrial function and the critical role mitochondria may play in neuronal cell death seen in ALS, we studied the clinical and biologic effects of a ketogenic diet in the G93A-SOD1 transgenic mice model of ALS.

## Results

### Clinical effect of the ketogenic diet in SOD1-G93A mice

**Figure 1:**
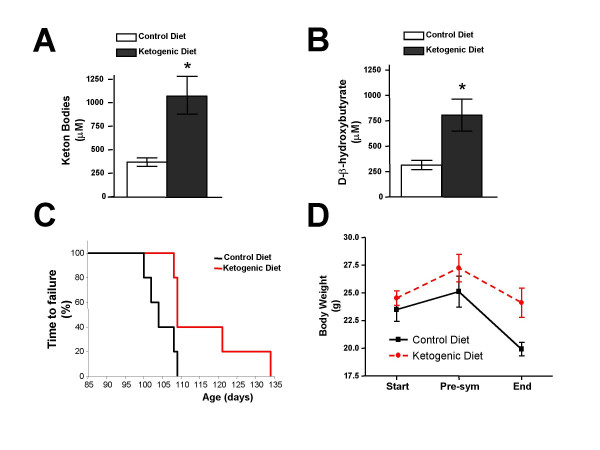
**Ketone levels, body weight motor function. **(A) The serum content of total ketone bodies (DHB and acetoacetate) in SOD1-G93A transgenic mice. (data = mean ± se; standard diet: n = 5, KD: n = 6; * - p < 0.05). (B) The serum content of DHB in SOD1-G93A transgenic mice. (data = mean ± se; standard diet: n = 5, KD: n = 6; * - p < 0.05). (C) Time to failure (50% baseline performance) in SOD1-G93A transgenic mice fed a KD compared to standard diet from 85 days to 136 days of age. (data = mean ± se; standard diet: n = 5, KD: n = 6; p = 0.027). (D) The effect of the KD on weight in SOD1-G93A mice at the beginning of treatment (Day 50), presymptomatic (Day 89) and endpoint of the study. (data = mean ± se; standard diet: n = 5, KD: n = 6_[AV3]; _p = 0.047).

A. Generation of Ketones: The KD fed mice showed a greater than 3.5 fold increase in the blood concentration of circulating ketone bodies (acetone, acetoacetate, and D-3-β hydroxybutyrate) compared to animals fed standard laboratory food (1056 ± 197 vs 360 ± 43 μM, p = 0.012) (Figure 1A). The principle form of ketone bodies is DHB, constituting more than 78% of blood ketones. DHB showed a greater than 2.5 elevation in the blood of KD fed animals compared to controls (806 ± 158 vs. 315 ± 46 μM, p = 0.023) (Figure 1B).

B. Measures of motor performance: Rotarod testing was used to determine the effect of the KD on the loss of motor function in SOD1-G93A transgenic mice. Time to failure analysis (Figure 1C) showed that through the period studied, KD fed animals maintained motor function longer than SOD1-G93 mice fed a standard diet. The last of the KD fed animals failed the rotarod endpoint of 50% of baseline performance (90 seconds) at day 134 compared to the standard diet fed group which failed at day 109 (p = 0.027). There was no statistically significant difference in the age at death between KD fed animals compared to SOD1-G93 transgenic mice fed a standard laboratory diet (133 ± 4 vs. 131 ± 4 days, p = 0.914)

C. Body weight: The weight of each mouse was measured at three points during the study period (Figure 1D). At the start of the study, animals assigned to the standard diet group had a mean weight of 23.5 g compared to 24.6 g in KD fed animals. During the presymptomatic stage (day 89), tested KD animals weighed more than standard feed mice (25.1 g and 27.8 g days respectively). At the endpoint of the study, weight was 19.9 g in the standard feed animals compared to 24.5 in the KD fed group. The difference in weight between treatment groups was not significant although a strong trend was present (p = 0.070). Interaction between diet and time was statistically significant (p = 0.047) indicating that the pattern of observed change of weight in KD animals was diet dependent. As expected, there was a statistically significant time effect with weight loss (p <0.001).

### The ketogenic diet protects against motor neuron death

**Figure 2:**
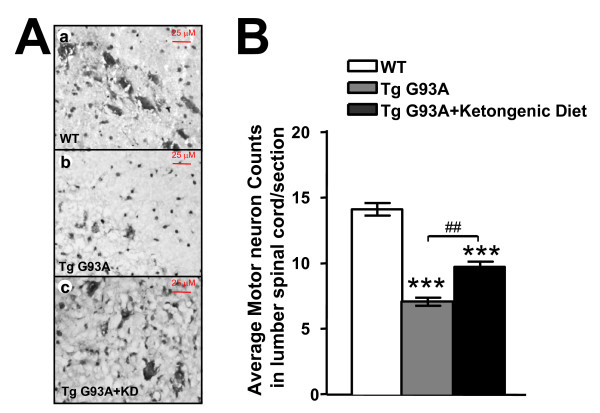
**Nissl-stained motor neuron count in the lumbar spinal cord. **The effect of the KD on Nissl-stained neuronal cell counts in SOD1-G93A transgenic mice at study endpoint. (A) Photomicrographs of representative Nissl-stained sections through the ventral horns of the lumbar spinal cord from wild-type (WT) (a), standard diet- (b) and KD (c) treated SOD1-G93A mice. Horizontal bar = 25 μm. (B) The counts of the large motor neurons in lumbar spinal cord of WT control and of SOD1-G93A treated with standard diet or KD (data = mean ± se; WT: n = 5, Tg G93A: n = 4, Tg G93A+KD n = 6; ## - p = 0.003, Tg G93A vs. Tg G93A+KD; *** - p < 0.001 vs. WT).

To determine whether the ketogenic diet protects against the motor neuron loss that accompanies the clinical symptoms of ALS, we counted the number of motor neurons in the lumbar spinal cord in age and gender matched WT and SOD1-G93A mice at the study endpoint (Figure 2A). Wild type animals had a mean of 13.594 ± 1.272 motor neurons per spinal cord section. In KD fed SOD1-G93A mice, there were significantly more motor neurons in the ventral horn compared to those in the SOD1-G93 mice fed standard feed at the endpoint of the study period (9.382 ± 1.125 vs 6.826 ± 0.607;(p = 0.03)) (Figure 2B). As expected, motor neuron counts in SOD1-G93 mice were significantly lower compared to WT irrespective of diet (p < 0.01).

### DBH effect on mitochondrial function in motor neurons from SOD1-G93A mice

**Figure 3:**
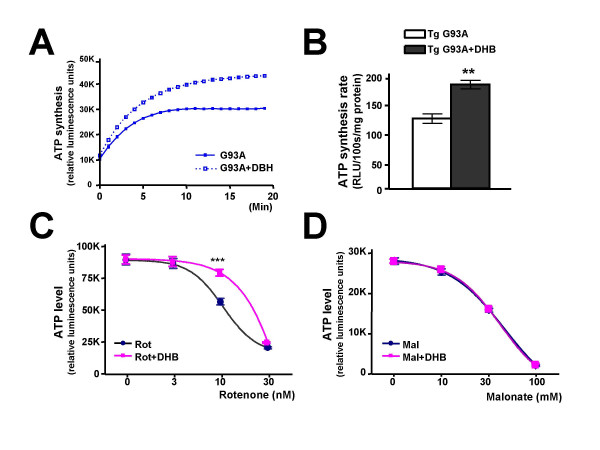
**Differential effect of DHB on mitochondrial activity. **(A) Representative examples of ATP synthesis measurements in spinal cord expressed as relative luminescence units (R.L.U.). (B) The rate of ATP synthesis in SOD1-G93A mitochondria purified from spinal cord (data = mean ± se; repeated measures, n = 9; ** - p < 0.001). (C) Effect of DHB on rotenone mediated cell viability. Primary mixed spinal cord neurons from SOD1-G93A mice were cultured in the presence or absence of DHB (5 mM) and exposed to mitochondrial Complex I inhibitor rotenone (data = mean ± se; Rot: n = 4, Rot+DHB: n = 4; *** - p < 0.001, Rot vs. Rot+DHB). (D) Absence of specific inhibition of DHB in SOD1-G93A cultures exposed to mitochondrial Complex II inhibitor malonate (data = mean ± se; Mal: n = 4, Mal+DHB: n = 4).

Mitochondrial dysfunction decreases ATP production in SOD1 mutant mice [14]. The cause is suspected to be abnormalities in the molecular complexes comprising the respiratory chain, particularly complex I [15]. The ketone body of highest content, DHB, corrects defects in mitochondrial energy generation by improving mitochondrial respiration and ATP production [13,16]. Our studies of purified mitochondria isolated from SOD1-G93A mice showed that addition of DHB increased ATP synthesis by 10000 relative luminescence units (RLU) within 12 minutes of exposure to substrate (Figure 3A). Within three minutes of adding DHB, the rate of ATP production showed a statistically significant increase compared to disease controls (198 ± 7 vs. 125 ± 9 RLU/100 s/mg protein, p < 0.001) (Figure 3B), thus confirming that DHB promotes ATP production in G93A-SOD1 transgenic mice.

To determine the particular complex that was dysfunctional in the respiratory chain motor neuron cultures were bathed in DHB (5 M) and then exposed to increasing doses of the complex I inhibitor rotenone (Figure 3B). Rotenone had no effect on ATP synthesis in motor neurons at concentrations less than 3 nM. As the dose of Rotenone increased to 10 nM, ATP production drops to 60% in untreated neurons but remains at 90% of ATP production in treated animals (57665 ± 1577 vs. 81485 ± 2545 RLU, p < 0.001) (Figure 3C). No detectable protection with respect to ATP generation by DHB was found in response to treatment with Complex II inhibitor, malonate in parallel cultures at any concentration tested (Figure 3D).

### DHB protects against cell death in motor neurons from G93-SOD1 transgenic mice

**Figure 4:**
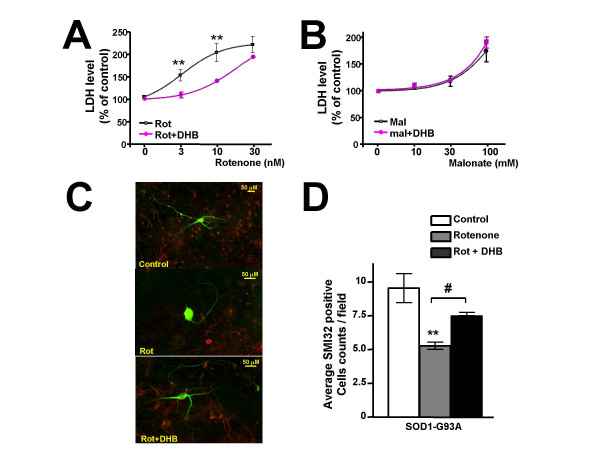
**Neuroprotective effects of DBH on spinal cord motor neurons. **(A) Effect of DHB on rotenone mediated toxicity. Primary spinal cord neurons from SOD1-G93A mice were cultured in the presence or absence of DHB (5 mM) and exposed to rotenone and cell toxicity measured by LDH activity released in the culture media expressed as a percentage of control (data = mean ± se; Rot: n = 4, Rot+DHB: n = 4; ** - p < 0.01, Rot vs. Rot+DHB). (B) Absence of neuroprotection of DHB in SOD1-G93A cultures exposed to malonate expressed as a percentage of control (data = mean ± se; Mal: n = 4, Mal+DHB: n = 4). (C) Photomicrographs of representative staining of motor neurons using SMI-32 antibody in control (top), rotenone (middle) and rotenone and DHB (bottom) treated spinal cord cultures. Motor neurons are SMI32 positive and characterized by their large cell bodies and numerous dendrites. (D) The effect of DHB on chronic mitochondrial toxicity by rotenone. Spinal cords neurons from SOD1-G93A were cultured in the presence or absence of DHB (5 mM) and treated with rotenone (3 nM) (data = mean ± se; Control: n = 4, Rotenone: n = 4, Rot+DHB: n = 4; # - p = 0.041, Rotenone vs. Rot+DHB; ** - p = 0.004, Rotenone vs. Control).

We next determined if cell death occurs as the result of reduced ATP production from complex I inhibition in motor neurons isolated from spinal cords of G93A-SOD1 transgenic mice. Rotenone applied to spinal cord motor neurons caused a dose dependent increase in the number of cells undergoing cell death by membrane lysis as measured by the amount of LDH in the medium (Figure 4A). DHB effectively blocks the effect of rotenone but there not malonate suggesting that DHB overcomes the inhibition of complex I, thereby reducing the occurrence of cell death (Figure 4B).

To test whether increased concentrations of LDH is actually associated with loss of motor neurons, immunohistochemically labeled motor neurons with SMI-32 were counted in three types of cell cultures: one obtained from normal mice and two from SOD1-G93A transgenic mice: one culture medium was treated with rotenone; the other with rotenone and DHB (Figure 4C). Neuron counts obtained from rotenone treated cultures from SOD1-G93 transgenic mice were significantly lower compared to neuron counts from DHB/rotenone treated cultures (5.5 ± 0.3 vs. 7.8 ± 0.1, p = 0.041) or cultures from wild type animals (5.5 ± 0.3 vs. 9.9 ± 1.1, p = 0.004) (Fig. 4D). Neuron counts obtained from rotenone+DHB treated cultures did not differ significantly from the counts obtained from wild type animals' cultures (7.8 ± 0.1 vs. 9.9 ± 1.1, p = 0.056). Therefore, DHB has a potential role in slowing the rate of cell death induced by complex I inhibition.

## Discussion

Our studies show that SOD1 G93A transgenic ALS mice on a KD showed significant alteration in the clinical manifestations and the biology of the disease. The preservation of motor performance in SOD1-G93A transgenic mice fed a KD diet was accompanied by a significant preservation of motor neurons in the lumbar spinal cord at the endpoint of the study. We observed a strong trend in weight change between the two groups suggesting KD fed animals gained weight faster during the presymptomatic phase and lost weight slower as the disease progressed. However there was a statistically significant relationship between diet and treatment, showing that the increase in weight in KD fed animals was dependent on their diet. We could not perform standard longevity analysis (Kaplan Meier) because the data failed the basic ANOVA requirements for normalcy and variance. However, there was no significant difference between treated and disease control groups at death. This most likely represents the loss of diet effect when weakening animals decrease food intake and therefore experience less of the benefit a diet based therapy would provide.

**Figure 5:**
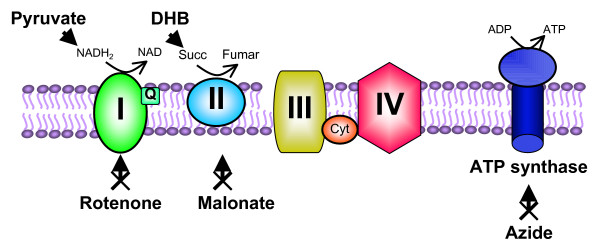
**Scheme 1** : Mitochondrial enzyme complexes of the respiratory chain and inhibition sites.

In primary spinal cord motor neuron cultures, neuroprotection is conferred by DBH against chronic mitochondrial inhibition induced by rotenone (Complex I) but not against malonate (Complex II)-mediated toxicity. This neuroprotective effect appears to be associated with increased ATP production in isolated spinal cord SOD1-G93A mitochondria when DHB is used as a substrate (Scheme 1, see Figure 5).

We found that DHB protected motor neurons derived from SOD1-G93A spinal cords *in vitro *from rotenone-mediated cell death. Rotenone decreases mitochondrial proton pumping and ATP production causing an increase in free radical production and subsequent neuronal cell death [17]. Kashiwaya et al. hypothesized that the neuroprotective effect of DHB might be related to its ability to restore the substrates for mitochondrial electron transport or its ability to decrease the production of reactive oxygen species (ROS) by modulating NAD/NADH and coenzyme Q level in the mitochondrial [16]. Moreover, Tieu et al. demonstrated that DHB may protect dopaminergic neurons against 1-methyl-4-phenyl-1,2,3,6-tetrahydropyridine (MPTP) mediated toxicity in an animal model of Parkinson's Disease. They proposed that DHB infusion overcomes the Complex I blockage by MPTP through the generation of succinate as an oxidative substrate not through reduction ROS [13]. Consistent with a Complex I bypass hypothesis, we found that DHB overcomes the toxicity elicited by rotenone in SOD1 G93A spinal cord motor neurons but is unable to overcome the inhibition of the mitochondrial Complex II by malonate.

We found that DHB, when used as an oxidative substrate, increases the rate of ATP production in mitochondria isolated from SOD1-G93A brain and spinal cord. This mechanism of action of DHB is consistent with studies by Sato et al., (1995) who found that ketones may increase the electric potential between mitochondria and cytoplasm and consequently the free energy of cytosolic ATP [18]. In the presence of DHB the efficiency of the working heart is increased by at least 25% due to an augmentation of contractibility and reduction of oxygen consumption. Ketone bodies appear to change the profile of energy metabolism and ketone bodies are metabolically more efficient than glucose or fats in mitochondrial ATP generation [18,19].

The neuroprotective effect of DHB in the ALS models *in vitro *and *in vivo *suggests that ketone bodies might have potential therapeutic benefit for patients with ALS and other neurodegenerative disorders [19]. The diet-induced elevation of blood ketones or an elevation of DHB to hyperketonemic concentrations might improve the mitochondrial defects by increasing mitochondrial function and ATP production. This reasoning has lead to a recent feasibility study in a small cohort of Parkinson's disease patients that concluded that although a placebo effect could not be excluded, resting tremor, freezing, balance, gait, mood, and energy levels were improved by the ketogenic diet [20]. With the possible advent of ketone esters for oral administration [19], clinical trials in ALS involving the use of ketone bodies as dietary supplements might be possible in the near future.

## Conclusion

This study provides experimental evidence showing that prophylactic treatment with a ketogenic diet may slow motor deterioration and protect motor neurons through a promoting energy production in the mitochondria of SOD1-G93A ALS mice. The neuroprotective effect by ketone bodies provides a new approach to potential treatment for ALS through a dietary intervention.

## Methods

### G93A transgenic mice and dietary conditions

Male SOD1-G93A mutant transgenic mice (stock #002297) were obtained from the Jackson Laboratory (Bar Harbor, ME) and bred in our transgenic mouse facility to generate SOD1-G93A mice and wild-type (WT) control littermates. Of the Twenty-seven SOD1-G93A transgenic ALS mice identified for this study, 11 males were selected. Only males were used because of the background and gender effects on survival in this mouse model of ALS [21]. At 50 days of age animals placed on either a ketogenic diet (caloric composition, fat 60%, carbohydrate 20%, protein 20%) or a standard rodent laboratory diet (fat 10%, carbohydrate 70%, protein 20%). Both diets contained equal percentages of cholesterol per gram (Research Diet, Inc. New Brunswick, NJ). Mice were housed on a 12 hour light, 12 hour dark cycle and allowed ad libitum access to food. Mice were weighed at the start of treatment, day 89 (pre-symptomatic) and at study endpoint. The study endpoint was defined as meeting any one of the following conditions: no spontaneous breathing or movement for 60 seconds with no response to pain; the animal is unable to right itself after 10 seconds following a push over; or complete hind limb paralysis.

Mice were sacrificed by cervical dislocation. Spinal cords were dissected and snap frozen and stored at -80°C. Blood was collected, and serum was obtained after 15 minutes of clotting and centrifugation at 4°C. The Institutional Animal Care Committee of Mount Sinai School of Medicine reviewed and approved all experimental protocols used in this study.

### Motor Function Assessment

SOD1-G93A mice were tested on the accelerating rotarod (7650 Ugo Basile Biol. Res. App., Comerio, Italy) as described previously [22]. Mice were given 5 days of practice to become acquainted with the rotarod and human handling. The examiner was blinded as to treatment group. At 85 days of life and continuing until death, the time the mouse could stay on the rotating rod was measured. Only animals that could successfully maintain balance for 180 sec for 2 successive days were included to ensure homogeneity of motor performance in all animals at onset. (n = 10: 5 KD; 5 standard feed). Testing was conducted during the last 4 hours of the day portion of the light cycle in an environment with minimal stimuli (noise, movement, changes in light or temperature).

### Histology and stereological analysis

For stereological analysis, 10 serial coronal sections (12 μm thick) were cut 350 μm apart through the lumbar (L3 to L5) spinal cord of each animal (WT controls; n = 5) and SOD1-G93A mice fed either the KD (n = 6) or standard diet (n = 4). The sections were mounted onto positively charged glass slides (Superfrost Plus, Fisher Scientific) and Nissel stained. Large (>25 μm), Nissl-stained neurons were counted within the ventral horns under a light microscope at a magnification of × 200. These counts were within a homogenous structure, making the tenets of stereology valid. Nissel-stained neurons were counted using the Neurolucida system at a magnification of × 250 in both ventral horn areas from six L3-L5 tissue sections of the spinal cord of each mouse, with size discrimination into diameter classes of >25 μm. All cells were counted from within the ventral horn below a lateral line across the spinal cord from the central canal. Correction for tissue section thickness was made in all specimens.

### Serum ketone measurement

There are three ketone bodies: D-β-3-hydroxybutyrate (DBH), acetone, and acetoacetate. The concentration of all three bodies represents the total concentration of ketone bodies in the blood. The concentration of ketone bodies in SOD1-G93A mice was determined using the Autokit 3-HB and Autokit Total ketone bodies respectively (Wako, Osaka, Japan), according to the manufacturer's instructions. Serum (3 μl diluted 5- or 10-fold) was incubated with 202.5 μl of reagent R1 in a 96 well plate, for 5 minutes at 37°C. Then 67.5 μl of reagent R2, was added for another 5 minutes and incubated at 37°C. The rate of Thio-NADH production was measured spectrophotometrically at 405 nm using a Dynatech MR5000 spectrophotometric 96 well plate reader (Coulton). A standard curve was established using two-fold dilutions of the Total Ketone Body calibrator (Wako) starting at 300 μmol/L. Samples were diluted 0, 3, 5 or 10 fold to fit within the standard curve which was linear over six 2-fold dilutions.

### Neuron cultures

Mixed spinal cord cultures were prepared according to Spalloni et al. [16,23]. Briefly, spinal cord cultures were prepared from E14 embryos dissected from a pregnant WT female mated with a SOD1-G93A male. Each spinal tube was dissected, removed from the meninges, and incubated for 10 min in 0.25% trypsin/EDTA at 37°C and then dissociated by gentle trituration with a fire-polished pasteur pipette. The resulting mixed neurons were plated on poly-D-lysine-coated 8 well chamber slides (Nalge Nunc Inc., NY) in D-MEM/F12 supplemented with 10% fetal bovine serum (FBS) and maintained at 37°C in a humidified atmosphere of 5% CO_2_. Two to three hours after plating, the medium was replaced with neurobasal medium supplemented with 2% B-27, 0.5 mM glutamine, and 1% penicillin/streptomycin.

### Toxicity experiments

Rotenone, malonic acid, sodium azide and DHB were obtained from Sigma-Aldrich, St Louis, MO. Ten-day-old neuron cultures were exposed to varying concentrations of rotenone or malonate with or without 5 mM DHB for 48 hours. Cell viability was measured by ATP production level using CellTiter-Glo^® ^luminescent cell viability assay kit according the manufacturer's instructions (Promega Corp Madison, WI).

Cytotoxicity was determined by the amount of lactate dehydrogenase (LDH) activity released in the cell culture media using CytoTox96^® ^Non-Radioactive Cytotoxicity Assay according the manufacturer's instructions (Promega Corp).

### Mitochondria preparations and ATP measurements

Mitochondria isolation and ATP measurements were performed using the mitochondria isolation kit and an Adenosine 5'-triphosphate (ATP) Bioluminescent Assay kits respectively according to the manufacturer's instructions (Sigma). Spinal cords from WT and Tg SOD1-G93A animals, 8 weeks of age, were dissected on ice and the tissue was quickly grinded in buffer A with a 3 ml Teflon pestle with 10 -15 strokes. After several centrifugation steps aimed at enriching mitochondria fraction, the final mitochondria pellet was resuspended in isolation buffer A (10 mM HEPES, pH7.5, 200 mM mannitol, 70 mM sucrose, 1 mM EDTA, and 1 mg/ml Albumin). Protein content was determined using the BioRad protein determination kit. For ATP generation measurements, assays were prepared in 100 μl total volume in a Fluorotrac 200 96 well plate (Nunc). Each assay contained buffer A (35 mM K-PO4, pH7.5, 10 mM Mg Acetate, 180 mM sucrose, 1 mM EDTA, 0.1% BSA, 50 mM pyruvate) with freshly added 100 μM ADP, 100 μM P^1^, P^5^-Di(adenosine) pentaphosphate, 150 μg/ml luciferin and 1.2 μg/ml luciferase, in the presence or absence of 5 mM DHB and the various inhibitors. The assays were initiated by adding a 10 μl volume of the mitochondrial preparation containing 30 μg of total isolated mitochondria. The luciferase light emission was recorded every minute for 10 min by Fusion™ Universal Microplate Analyzer (Perkin Elmer, MA).

### Immunocytochemistry

The 10-day-old neuron cultures were treated with drug for 24 hours and cultured with fresh drug free culture media for another 48 hours with or without 5 mM DHB. The cells were fixed with 4% paraformaldehyde and processed to immunocytochemistry with anti-NSE and anti-SMI32 double staining. Total neurons were identified as NSE positive neurons, and motor neurons were regarded as SMI32 positive neurons. The SMI32 positive neurons were counted microscopically at a magnification of × 250 in as least 5 randomly selected fields. The examiner was blind to the treatment.

### Statistical analysis

Statistical analyses were performed using SigmaStat (version 3.0, SPSS Inc., Chicago, IL). Independent measures t-tests were used to compare endpoint serum contents of DHB and total ketone bodies in control vs. ketogenic diet group. Repeated measures t-test was used to compare ATP synthesis rate data obtained from neuron cultures incubated with and without addition of DHB. Deterioration of motor function (rotarod testing) was assessed by the Kaplan-Meier survival analysis (Mantel-Cox log rank test) with "failure" defined as inability of an animal to achieve at least 50% of its baseline motor performance. This endpoint was chosen because it is a measure that is distinctly related to entry criteria, is the midline of an inexorable decline in strength, and is not influenced by observer bias and animal adaptive skills used when the animal is very weak (e.g. holding on to the rotating rod without walking). A nonparametric time to failure analysis was chosen since a two-way repeated measures ANOVA could not be performed because the data failed both normality and equal variance tests. Effect of ketogenic diet on longevity was also assessed by the Kaplan-Meier survival analysis (Mantel-Cox log rank test) with "failure" defined as death of an animal. Animals' body weight data were analyzed by a two-way repeated measures ANOVA with Huynh-Feldt correction. Motor neuron counts data and SMI32 positive neuron counts data from immunochemical evaluation were analyzed by one-way ANOVAs. Data on ATP and LHD levels in the presence of rotenone and malonate were analyzed by two-way ANOVAs. ANOVA tests were followed, when significant, by the Student-Newman-Keuls multiple comparison tests. Student-Newman-Keuls test was chosen for post hoc multiple comparisons due to its generally higher sensitivity compared to the Bonferroni test. In all tests, results with probability values less than 0.05 were considered statistically significant. Presented data are shown as mean ± se, unless otherwise noted.

## Abbreviations

ALS: amyotrophic lateral sclerosis

KD: ketogenic diet

DBH: D-β-3 hydroxybutyrate

SOD: Cu/Zn superoxide dismutase

RLU: relative luminescence units

LDH: lactate dehydrogenase

ROS: reactive oxygen species

## Competing interests

The author(s) declare that they have no competing interests.

## Authors' contributions

ZZ and DM: contribute to project conception and experimental design, acquisition, analysis and interpretation of data, also involved in drafting and revising the manuscript.

DJL: involved in project conception and design, data interpretation, and manuscript revision.

AV: statistical analysis and manuscript revision

LH: contribute to project conception and experimental design, animal behavior analysis.

JS and NH: contribute to animal care and behavior analysis.

JW: carried out the ketone body measurement.

TM: carried out the immunocytochemistry study.

GMP: involved in project conception and experimental design, data interpretation, manuscript preparation and final approval.
